# Cerebrospinal Fluid Proteomics in Down Syndrome Identifies Common and Unique Pathological Changes Compared to Late‐Onset and Autosomal Dominant Alzheimer’s Disease

**DOI:** 10.1002/alz.089529

**Published:** 2025-01-09

**Authors:** Shijia Bian, Laia Montoliu‐Gaya, Eric B. Dammer, Mathias Sauer, Daniel Alcolea, Kaj Blennow, Henrik Zetterberg, Juan Fortea, Erik C.B. Johnson

**Affiliations:** ^1^ Rollins School of Public Health, Emory University, Atlanta, GA USA; ^2^ University of Gothenburg, Mölndal Sweden; ^3^ Emory University Center for Neurodegenerative Disease, Atlanta, GA USA; ^4^ University of Gothenburg, Göteborg, Göteborg Sweden; ^5^ Hospital de la Santa Creu i Sant Pau ‐ Biomedical Research Institute Sant Pau ‐ Universitat Autònoma de Barcelona, Barcelona Spain; ^6^ Institute of Neuroscience and Physiology, The Sahlgrenska Academy at the University of Gothenburg, Mölndal Sweden; ^7^ Institute of Neuroscience and Physiology, Sahlgrenska Academy at the University of Gothenburg, Gothenburg Sweden; ^8^ Sant Pau Memory Unit, Hospital de la Santa Creu i Sant Pau, Biomedical Research Institute Sant Pau, Universitat Autònoma de Barcelona, Barcelona Spain; ^9^ Emory University School of Medicine, Atlanta, GA USA; ^10^ Emory Goizueta Alzheimer's Disease Research Center, Atlanta, GA USA

## Abstract

**Background:**

Nearly all people with Down Syndrome (DS) develop Alzheimer’s dementia (AD) by the 7^th^ decade of life. However, whether the alterations in fluid biomarker levels associated with DS follow the same pattern to those observed in other forms of AD is not well understood.

**Method:**

We used mass spectrometry‐based proteomics to measure 1116 proteins in cerebrospinal fluid (CSF) across euploid controls (n=130), sporadic late‐onset AD (LOAD, n=89), asymptomatic DS (n=117), prodromal DS (n=57), and dementia DS (n=80) cases, and compared the protein changes observed in DS to those in LOAD and to those recently described in autosomal dominant AD (ADAD).

**Result:**

We observed both common and unique CSF proteomic alterations in DS compared to LOAD and ADAD. Common changes included increased levels of 14‐3‐3 proteins, glucose metabolism, immune activation, and decreased levels in neurosecretory and neuropentraxin synaptic markers. Common changes were strongly correlated to tau and Aβ pathology, and like ADAD, most CSF protein changes occurred prior to the onset of cognitive symptoms. Compared to LOAD, DS showed markedly early elevations in complement, immunoglobulins, extracellular matrix and plasma proteins, suggesting early dysfunction in immune‐related processes and blood‐brain barrier function. These DS‐related changes were observed to occur prior to the onset of Aβ or tau pathology. In contrast to the progression observed in ADAD, in DS neurofilament light was increased prior to total tau levels, which were in turn increased prior to pTau levels, suggesting relatively earlier white matter and axonal dysfunction in DS. Neuronal and neurosecretory proteins were also decreased earlier in DS than in ADAD, consistent with early neuronal dysfunction in DS. Compared to LOAD, DS demonstrated more significant elevations in endosome/lysosome markers in symptomatic phases of the disease.

**Conclusion:**

Early pathological alterations in immune function and the blood‐brain barrier associated with trisomy 21 may contribute to the onset of AD pathology in DS. While most pathological alterations are shared between DS, ADAD, and LOAD, the temporal ordering of these pathological changes in DS has unique features.

